# Prevalence of normal weight central obesity among Thai healthcare providers and their association with CVD risk: a cross-sectional study

**DOI:** 10.1038/srep37100

**Published:** 2016-11-16

**Authors:** Lakkana Thaikruea, Jiraporn Thammasarot

**Affiliations:** 1 Community Medicine Department, Faculty of Medicine, Chiang Mai University, Thailand; 2 Health Promotion Center, Maharaj Nakorn Chiang Mai Hospital, Faculty of Medicine, Chiang Mai University, Thailand

## Abstract

This study aims to determine the prevalence of health personnel with normal weight central obesity and to investigate whether this group had higher cardiovascular disease (CVD) risk factors than those of the people with normal weight and without central obesity. A waist-to-height ratio was calculated as waist circumference (at umbilical level) in cm divided by height in cm. The central obesity cut-off level was 0.5. The body mass index was calculated as weight in kg divided by height in meters squared. The obesity cut-off level was 25 kg/m^2^. The prevalence of health personnel with normal weight central obesity was 15.4% (499 out of 3235). When compare this group to 1787 health personnel who had normal weight and without central obesity, they were 2.03 times (95% CI of adjusted OR; 1.62 to 2.54) more likely to have at least one CVD factor. The waist-to-height ratio cut-off value of 0.5 can be used as a self-assessment tool for central obesity without the need for a standard measuring tape. It is feasible to be implemented in screening or self-monitoring for the general population.

The World Health Organization has reported that the relative distribution of excess fat could be used to determine risks of cardiovascular disease more effectively than its total amount^1^. Computed tomography (CT) and magnetic resonance imaging (MRI) have been used to classify fat distribution^2^,^3^. A preferential deposition of fat in the internal visceral region is classified as an apple shape, which has been deemed unhealthy. An external subcutaneous fat deposition has been classified as the pear shape, which has been assessed as being healthier^4^. CT and MRI scans are expensive and are not really feasible in a developing country. Waist circumference (WC) has been proposed as an anthropometric indicator for obesity^5^,^6^,^7^. The cut-off values are varied and are lower in Asians than those of Europeans^7^,^8^,^9^. There are different protocols of WC measurement, including measuring at umbilical level, the narrowest waist, at the midpoint between the lowest rib and the iliac crest, and immediately above the iliac crest^10^,^11^,^12^. These measurement methods produce different WC values. Furthermore, WC values also vary by gender^13^,^14^,^15^. Thus, different cut-off levels of WC are not practically appropriate indicators for CVD risk factors. A more simple anthropometric measurement used for self-assessment or monitoring of CVD risk factors is proposed, which is WC weighted by each individual’s height or ‘waist-to-height ratio (WHtR)’^16^. The cut-off value of WHtR indicating increased health risk reported by studies in different races and age groups is above 0.5. These races included people from Australia, India, Korea, China, and Thailand^13^,^17^,^18^,^19^,^20^. A large cohort of the British population showed that WHtR was a better predictor of mortality than BMI^21^,^22^. The WC commonly used in calculating WHtR is measured at the midpoint between the lower costal margin and superior iliac crest^15^. However, the level of precision is more difficult and also less practical than measurement at an umbilical level. The Thai Ministry of Public Health launched a campaign addressing obesity prevention “keep your waist circumference lower than half of your height” using WC measurement at umbilical level. These two measurements of WC gave different results and led to bias. In Japan, the differences had a range of 3.9 cm among men to 12.6 cm among women^14^. Experts from the UK proposed a WHtR cut-off value of 0.5 in adults, with a similar ratio in children and adolescents^4^,^18^. A systematic review found an appropriate cut-off level of a 0.5 WHtR in various ethnic groups and indicated that WHtR could be used as a screening tool for predicting CVD and diabetes^23^. The study involving health personnel in Thailand also found a WHtR cut-off value of 0.5 associated with CVD risk factors^20^. Although the campaign of “keep your waist circumference lower than half of your height” has been promoted for many years in Thailand, health personnel still use BMI for screening and monitoring in their services. This practice is our concern because the sector of the population with weight within normal limits (defined by BMI) and central obesity (defined by WHtR) are missed. This group with normal weight central obesity had a higher mortality than people with normal weight and without central obesity^24^. Recently, there is evidence which supports the fact that central obesity increases mortality risk and CVD mortality rates^25^,^26^. The collaborative analysis of 58 prospective studies found that subgroups of WC were associated with increased mortality risk for a given BMI category in prediction models^26^. Another study found that central obesity measurement was more highly associated with total and CVD mortality rates than with BMI^25^. On analysis the data of individual-patients with coronary artery disease showed that people in the top tertile of central obesity measures who had normal BMI had the highest total mortality rate^25^. A study in the UK showed that 9% of men and 11% of women who were deemed low risk by their BMI were at risk according to WHtR because they had central obesity^4^. Unfortunately, the recent suggested management on obesity guidelines by the American Heart Association/American College of Cardiology/The Obesity Society 2013 does not clearly recommend WHtR measurement due to limited evidence^27^.

WHtR requires WC measurement for calculation. Although the values of WC vary by measurement methods and gender, WHtR does not different much^13^. WHtR using WC measurement at umbilical level has a greater correlation with present trunk fat and a higher sensitivity than those of WHtR using other WC measurements. The UK study used WC measurement at the midpoint between the lower costal margin and iliac crest for WHtR calculation (WHtRM)^4^. Thaikruea *et al.* conducted a study involving the health personnel of Faculty of Medicine, Chiang Mai University (CMU), Thailand. They found that although the value of WC measured at umbilical level (WHtRU) was different from WHtRM in each individual but the WHtR calculations delivered by both WC methods were not significantly different. They also found that WHtRU was a better indicator for CVD risk factors than WHtRM^20^. From a prevention and health promotion perspective, WHtRU may be used for community screening and in self-assessment for central obesity rather than BMI. The WHtRU is simple and does not require height and WC measurements in centimetres or inches. The study aims to estimate the prevalence of normal weight central obesity among healthcare personnel and to investigate whether this group were of higher risk of CVD factors than those of the people with normal weight and without central obesity.

## Results

### Demographic

3235 health personnel participated in the study. The majority of them were female (76.65%) with a mean age of 40.20 years (SD 10.73 years). 64.61% had Bachelor or higher. 15.4% of the health personnel had a classification of a normal weight central obesity (WHtRU of at least 0.5 and a BMI below 25 kg/m^2^). 55.2% of health personnel had both a BMI and waist circumference within normal limits (WHtRU below 0.5 and BMI below 25 kg/m^2^) (Table 1).

### Risk group by waist-to-height ratio

42.9% of the health personnel who were in the increased risk and very high risk groups (Table 2).

### CVD risk factors by waist-to-height ratio and body mass index

The group with WHtRU of at least 0.5 and a BMI of at least 25 kg/m^2^ had the highest values of cholesterol, TG, LDL, and FBG compared to those of other groups. This group had the lowest HDL compared to those of other groups (Table 3). They had higher cholesterol, TG, LDL, and FBG than those of the group with WHtRU below 0.5 regardless of their BMI (Table 3).

The prevalence of CVD risk factors among the normal weight central obesity were high. The normal weight central obesity group was more likely to have CVD risk factors than those of the normal weight group. The relative risks ranged from 1.27 (95% CI: 1.18 to 1.37) to 3.20 (95% CI: 1.88 to 5.43) (Table 4).

### Relationship between CVD risk factors and waist-to-height ratio and body mass index

When adjusted for age and gender, people in the normal weight central obesity group were more likely to have a CVD risk factor than those of the normal weight group (Table 5). They were also more likely to have a lower HDL than those of reference. Over all, the normal weight central obesity group were 2.03 times (95% adjusted OR; 1.62 to 2.54) more likely to have at least one CVD risk factor compared to the normal weight group (Table 5).

## Discussions

WC shows a correlation with intra-abdominal adipose tissue and subcutaneous abdominal adipose tissue, which is related to diabetes^5^,^6^,^18^,^28^,^29^. Thus, WC is a better anthropometric marker of intra-abdominal adipose tissue than is BMI^30^. There are different protocols of WC measurement, including measuring at umbilical level, the narrowest waist, at midpoint between the lowest rib and the iliac crest, and immediately above the iliac crest^12^,^30^. These measurement methods produce different WC values. Furthermore, WC values also vary by gender^13^,^14^,^15^. Thus, different cut-off levels of WC are not practically appropriate indicators for the assessment of CVD risk factors^15^. A more simple anthropometric measurement used for self-assessment or monitoring of CVD risk factors is proposed, which is WC as a ratio to each individual’s height or ‘waist-to-height ratio’^16^. Our study used WHtR for the assessment of CVD risk factors. Although values of WC vary by measurement method and gender, WHtR does not vary much when using different WC measurement methods. Our study used WC measurement at umbilical level because it has a higher correlation with present trunk fat and a high level of sensitivity^13^,^20^. It is also easy to measure and understandable for laypeople. The cut-off value of WHtR indicating increased health risk was found to be 0.5, which is supported by studies in different races and age groups^4^,^17^,^20^,^31^,^32^.

Our study showed that there were 42.9% of CMU health personnel who were in the increased risk and very high risk groups. The health personnel who had WHtRU of at least 0.5 but had a BMI below 25 kg/m^2^ (in the normal weight central obesity group) were more likely to have CVD risk factors than those of the health personnel who had WHtRU below 0.5 and BMI below 25 kg/m^2^. This group, which was about 15% of health personnel, was missed using the BMI definition method. They might perceive that they had no risk, although they had. A study among Thai people found that individuals with a BMI at low as 23 kg/m^2^ were associated with CVD risk factors^20^. The normal weight central obesity who were at risk was 25% of the UK population^31^.

Our study found that the prevalence of CVD risk factors among the normal weight central obesity were high. When adjusted for age and gender, the normal weight central obesity group were more likely to have hypertension and to have high cholesterol, TG, LDL, and a FBG higher than those of a reference group. The adjusted OR ranged from 1.27 (95% CI = 1.18–1.37) of total cholesterol to 3.20 (95% CI = 1.88–5.43) of diabetics. Over all, the normal weight central obesity group were two times more likely to have at least one CVD risk factor than those of reference group. Other studies involving systematic reviews and meta-analyses have found that WHtR predicted CVD risk factors better than WC^23^,^32^,^33^. A study which included more than 300,000 adults from several ethnic groups supported our findings. It showed that WHtR had a higher discriminatory power than WC in indicating diabetes, hypertension, CVD and all outcomes regardless of gender. Furthermore, the rank order from highest to lowest for AUC was always WHtR, WC, then BMI. The findings also showed that Asian people had more positive results of WHtR and a higher correlation than Caucasians^33^. The study in Singapore based on an Asian employee health screening in 2012 had similar findings to our study. The research team also used WC measurement at umbilical level and defined a WHtR cut-off value of 0.5 for central obesity similar to data used in our study. They found that a WHtRU of at least 0.5 could identify the highest proportion of all the CVD risk factors regardless of gender, which supported our findings^34^.

The study in the UK described central obesity and peripheral obesity as shapes of apples and pears, terms which are easy for the general population to understand^4^. An apple shape reflects an internal visceral fat deposition. A pear shape reflects external subcutaneous fat deposition. Although, Thai people are familiar with apples, pear and their shape may be less well-known. Therefore, for health risk communication, the phase of ‘keep waistline less than half of your height’ is easy to understand by laypeople in Thailand. Anthropometric measures usually practically enable the assessment of CVD risk factors with between 60% and 70% accuracy in practice and also in population-based screening measures^33^. However this study of WHtRU in CMU health personnel has 73.6% sensitivity, 67.6% specificity, and 69.1% corrected classification of having at least two risk factors of CVD. Thus, WHtRU has good accuracy in screening measures^20^.

Due to the nature of a cross-sectional study design, the identification of risk cannot be guaranteed in our study. However, other prospective and meta-analysis studies support the association and prediction of WHtR for CVD risk factors and health related outcomes. An 11-year prospective study among over 45,000 women below the age of 60 found that WHtR was the strongest predictor of strokes in women when compared to WC and waist-to-hip ratio, whilst BMI was not a good predictor. Although the location of visceral adipose tissue (VAT) measurement affected an association magnitude of the metabolic syndrome definitions, VAT still remained significantly associated with metabolic syndrome regardless of measurement location. Also a clinical interpretation was not changed regardless of different measurement methods or metabolic syndrome definitions^21^,^22^,^32^,^33^,^34^. This means our findings might not be affected much by this condition. The strengths of our study include the large sample size (more than 3,000 persons), the conduction of the investigations by trained health personnel, and the standardisation of laboratory investigations by the Medicine Faculty.

## Conclusion

About 42.9% of our study population had an increased risk or a very high risk of CVD. A screening tool sensitive to visceral/trunk fat rather than body mass is more appropriate. BMI misclassified 15.4% of central obesity as non-obesity. WHtRU was more effective than BMI and WC as a screening method for CVD risk factors. The cut-off value of 0.5 can be used as a self-assessment tool for central obesity without the requirement of a standard measuring tape because it is just half the length compared to individual height^20^. It is worth to study about implementing this method in screening or self-assessment for the population who have normal weight central obesity. Further research is needed to evaluate the effectiveness of WHtRU in public health screening among the general population who are overweight.

## Methods

### Data

The Medicine Faculty of CMU provided a non-communicable disease screening for all health personnel during 2012 to 2013. All health personnel were eligible. Sample size estimation was 491 persons based on expected prevalence of a normal weight central obesity 15%, acceptable error margin of 3%, confidence level of 95%, and design effect 1.0. The participants were interviewed, had a physical examination, and had blood drawn for laboratory testing. Anthropometric measurements included height, weight, and waist circumference. A portable stadiometer and an electronic scale were used for measuring standing height (without shoes) in centimetres (cm) and weight in kilograms (kg). A standard measuring tape was used to measure horizontal waist circumference at the umbilical level. Trained investigators performed all measurements. Three blood pressure readings were taken five minutes apart using an ADC ^®^ Digital E-Sphyg and two non-mercury sphygmomanometers with appropriate cuff size. These machines were calibrated every two months. Venous blood samples were drawn and processed at the Central Diagnostic Laboratory in CMU Hospital for a fasting blood glucose and lipid profile.

The BMI was calculated as weight in kg divided by height in meters squared (kg/m^2^). The obesity cut-off level was 25 kg/m^2^. The waist-to-height ratio (WHtR) was calculated as WC in cm divided by height in cm. Based on the Thai Ministry of Public Health, the obesity cut-off level was at least 0.5. Health personnel who had WHtR of at least 0.5 and BMI below 25 kg/m^2^ were defined as the ‘normal weight central obesity group’. Health personnel who had WHtR below 0.5 and BMI below 25 kg/m^2^ were normal weight, non-central obesity group who were used as the reference group.

Risk factors for cardiovascular disease (CVD) included hypertension, diabetes, and dyslipidemia. High blood pressure or hypertension was present if each individual was a known case of HT or had a systolic blood pressure of at least 140 and/or a diastolic blood pressure of at least 90 mmHg^35^. Diabetes was present if each individual was either diagnosed by physician or had a fasting blood glucose of at least 126 milligram per decilitre (mg/dl)^36^. Dyslipidaemia was defined as present if the individual had any one of the following conditions: 1) known case of dyslipidemia, or 2) one of these abnormal lipid profiles, which were total cholesterol of at least 200 mg/dl, triglycerides (TG) of at least 150 mg/dl, low density lipoprotein (LDL) of at least 160 mg/dl, or high density lipoprotein (HDL) below 40 mg/dl in men and below 46 mg/dl in women^20^,^37^.

All methods were carried out in accordance with relevant guidelines and regulations. All experimental protocols were approved by the Ethics Committee of the Faculty of Medicine, CMU (No. 069/2012). Informed consent was obtained from all subjects.

### Statistical analysis

Descriptive analyses included proportion, mean +/− standard deviation (SD), or median (range) depending on data distribution. Univariate analysis includes relative risk (RR) with 95% confidence interval (95% CI), Chi Square test, and Kruskal-Wallis test. Multivariate analysis included multiple logistic regression (adjusted odds ratio with 95% CI). A p-value of below 0.05 was considered as statistically significant. Data management and analyses were performed using Epi Info for Windows version 3.5.4 (Centers for Disease Control and Prevention, Atlanta, GA) and STATA version 11 (Statacorp LP. College Station, TX).

## Additional Information

**How to cite this article**: Thaikruea, L. and Thammasarot, J. Prevalence of normal weight central obesity among Thai healthcare providers and their association with CVD risk: a cross-sectional study. *Sci. Rep.* **6**, 37100; doi: 10.1038/srep37100 (2016).

**Publisher's note**: Springer Nature remains neutral with regard to jurisdictional claims in published maps and institutional affiliations.

## Figures and Tables

**Table 1:** Demographic characteristics of health personnel, Faculty of Medicine, Chiang Mai University.

Characteristics	Frequency	Percent
Gender		
Female	2491	76.84%
Male	751	23.16%
Total	3242	100.00%
Education		
Primary School	52	1.61%
Junior high school	177	5.48%
Secondary high school	913	28.29%
Bachelor	1703	52.77%
Higher than Bachelor	382	11.84%
Total	3227	100.00%
Marital Status		
Single	1390	43.07%
Married	1477	45.77%
Separate	96	2.97%
Divorced	179	5.55%
Widow	77	2.39%
Unknown	8	0.25%
Total	3227	100.00%
Hypertension diagnosis		
No	2360	85.63%
Yes	396	14.37%
Total	2756	100.00%
Diabetic diagnosis		
No	1873	95.90%
Yes	80	4.10%
Total	1953	100.00%
Dyslipidemia diagnosisPercent		
No	1106	65.14%
Yes	592	34.86%
Total	1698	100.00%
WHtRU and BMI*		
WHtRU at least 0.5 and BMI at least 25 kg/m^2^	890	27.52%
WHtRU below 0.5 and BMI at least 25 kg/m^2^	59	1.82%
WHtRU at least 0.5 and BMI below 25 kg/m^2^*	499	15.42%
WHtRU below 0.5 and BMI below 25 kg/m^2^	1787	55.24%
Total	3235	100.00%

^*^WHtRU waist-to-height ratio using waist circumference at umbilical level BMI body mass index.

**Table 2:** Health personnel and risk categorised by waist-to-height ratio.

Group	Frequency	%
Very high risk (WHtRU* at least 0.6)	182	5.6
Increased risk (WHtRU at least 0.5 and below 0.6)	1207	37.3
Non-increased risk (WHtRU below 0.5)	1846	57.1
Total	3235	100.0

^*^WHtRU waist-to-height ratio using waist circumference at umbilical level.

**Table 3:** CVD risk factors in each group of health personnel categorized by waist-to-height ratio and body mass index.

Group	CVD risk factors	
	Mean	Standard Deviation
Total Cholesterol (mg/dl)		
WHtRU >= 0.5 and BMI < 25	210.75	36.58
WHtRU < 0.5 and BMI < 25	201.50	36.62
Triglyceride (mg/dl)		
WHtRU >= 0.5 and BMI < 25	113.40	83.96
WHtRU < 0.5 and BMI < 25	79.58	57.84
Low density lipoprotein (mg/dl)		
WHtRU >= 0.5 and BMI < 25	135.28	32.60
WHtRU < 0.5 and BMI < 25	126.67	34.22
High density lipoprotein (mg/dl)		
Men		
WHtRU >= 0.5 and BMI < 25	49.94	10.17
WHtRU < 0.5 and BMI < 25	55.24	12.85
Women		
WHtRU >= 0.5 and BMI < 25	60.68	13.39
WHtRU < 0.5 and BMI < 25	63.11	12.39
Fasting blood glucose (mg/dl)		
WHtRU >= 0.5 and BMI < 25	92.81	16.15
WHtRU < 0.5 and BMI < 25	87.94	12.41

^*^WHtRU waist-to-height ratio using waist circumference at umbilical level.

^†^BMI body mass index.

**Table 4:** Comparing prevalence of CVD risk factors between health personnel with normal weight central obesity and health personnel with normal weight and without central obesity.

Group*	CVD risk factors†					
	Yes	No	Total	Relative Risk	Lower limit	Upper limit
	Hypertension					
NWCO	235	264	499	1.81	1.60	2.04
Row%	47.09%	52.91%	100.00%			
NW	466	1321	1787			
Row%	26.08%	73.92%	100.00%			
	Diabetics					
NWCO	25	474	499	3.20	1.88	5.43
Row%	5.01%	94.99%	100.00%			
NW	28	1759	1787			
Row%	1.57%	98.43%	100.00%			
	Cholesterol					
NWCO	341	158	499	1.27	1.18	1.37
Row%	68.34%	31.66%	100.00%			
NW	956	827	1783			
Row%	53.62%	46.38%	100.00%			
	LDL					
NWCO	188	311	499	1.58	1.37	1.82
Row%	37.68%	62.32%	100.00%			
NW	426	1361	1787			
Row%	23.84%	76.16%	100.00%			
	Triglyceride					
NWCO	185	314	499	1.95	1.68	2.26
Row%	37.07%	62.93%	100.00%			
NW	340	1447	1787			
Row%	19.03%	80.97%	100.00%			
	HDL					
NWCO	160	339	499	1.73	1.47	2.03
Row%	32.06%	67.94%	100.00%			
NW	331	1456	1787			
Row%	18.52%	81.48%	100.00%			
	Had at least one CVD risk factor					
NWCO	343	156	499	1.54	1.42	1.67
Row%	68.74%	31.26%	100.00%			
NW	798	989	1787			
Row%	44.66%	55.34%	100.00%			

^*^NWCO normal weight central obesity.

NW normal weight without central obesity.

^†^CVD risk factors included 1) Hypertension = known case or high blood pleasure 2) Diabetics = known case or fasting blood glucose at least 126 mg/dl 3) cholesterol = Total cholesterol at least 200 mg/dl 4) LDL = Low density lipoprotein at least 160 mg/dl 5) Triglyceride = Triglyceride at least 150 mg/dl 6) HDL = High density lipoprotein below 40 mg/dl in men and below 46 mg/dl in women

**Table 5:** Comparison of CVD risk factors between normal weight central obesity group and normal group.

CVD risk factors	Adjusted Odds Ratio (95% CI)*
Hypertension or high blood pleasure	2.06 (1.65–2.55)
Diabetic or fasting blood glucose at least 126 mg/dl	1.90 (1.42–2.56)
Total cholesterol at least 200 mg/dl	1.40 (1.13–1.75)
Low density lipoprotein at least 160 mg/dl	1.36 (1.09–1.71)
Triglyceride at least 150 mg/dl	1.50 (1.17–1.92)
High density lipoprotein below 40 mg/dl in men and below 46 mg/dl in women	1.45 (1.15–1.84)
Had at least one CVD risk factor	2.03 (1.62–2.54)

^*^Adjusted for age and gender with p-value < 0.01.
